# Basilar Artery Lateral Displacement May Be Associated with Migraine with Aura

**DOI:** 10.3389/fneur.2018.00080

**Published:** 2018-02-21

**Authors:** Cen Zhang, John A. Detre, Scott E. Kasner, Brett Cucchiara

**Affiliations:** ^1^Department of Neurology, New York University, New York, NY, United States; ^2^Department of Neurology, University of Pennsylvania, Philadelphia, PA, United States

**Keywords:** magnetic resonance imaging, migraine with aura, vertebrobasilar artery, migraine, posterior circulation

## Abstract

**Objective:**

The objective of this study is to determine whether structural features of the vertebrobasilar arterial system are related to migraine.

**Background:**

Alterations in cerebral vascular structure and function have been associated with migraine, possibly mediated by hypoperfusion and/or endothelial dysfunction triggering cortical spreading depression. Vessel tortuosity, in particular, has been associated with both altered hemodynamics and endothelial function. Symptoms of migraine with aura (MWA) often localize to the occipital cortex, and evidence supports the localization of a migraine generator to the brain stem, suggesting that the vertebrobasilar system may be of particular relevance.

**Methods:**

We performed a *post hoc* exploratory analysis of data collected in a prospective, observational, case-control study enrolling MWA, migraine without aura (MwoA), and control subjects in a 1:1:1 ratio. 3 T high-resolution MR angiography was used to assess vascular structure, and arterial spin-labeled perfusion MRI to measure interictal cerebral blood flow (CBF). White matter lesions were assessed using T2/FLAIR. Vertebral and basilar artery (BA) diameters and BA total lateral displacement were measured.

**Results:**

162 subjects were included (52 control/52 MWA/58 MwoA). Mean age was 33 ± 6 years, and 78% were female. BA diameter was similar across groups (3.6 ± 0.6 mm in all 3 groups). BA displacement was similar in MwoA (5.1 ± 3.0 mm) and controls (4.9 ± 3.1 mm), but tended to be greater in MWA (6.3 ± 3.8 mm, *p* = 0.055 vs. controls). BA displacement increased with age (*p* < 0.001) was greater in men vs. women (6.6 ± 4.2 vs. 5.1 ± 3.0, *p* = 0.02) and with increased migraine frequency (*p* = 0.03). In multivariate analysis, BA displacement was significantly greater in MWA subjects (*p* = 0.02), with older age (*p* = 0.003), and in men (*p* = 0.046). In regression analysis adjusted for age and sex, BA displacement remained significantly greater with increasing migraine frequency (*p* = 0.02). There was no association between BA displacement and interictal posterior cerebral artery territory CBF or overall white matter lesions.

**Conclusion:**

BA lateral displacement may be associated with MWA as well as headache frequency. This association does not appear to be mediated by cerebral hypoperfusion.

## Introduction

Multiple lines of evidence support an interaction between altered cerebrovascular structure and function and migraine. Laboratory models have shown that microembolism, cerebral ischemia, and endothelial dysfunction can trigger cortical spreading depression, felt to be the chief mechanism underlying migrainous aura (1, 2). Measures of arterial stiffness are altered in migraine subjects, as are biomarkers of endothelial dysfunction (3, 4). Epidemiologic data support an increased risk of ischemic stroke in subjects with migraine, particularly migraine with aura (MWA), and imaging studies show an increased prevalence of white matter lesions on MRI and structural variants in the circle of Willis in migraine (5–8). A recent pooled genetic study examining almost 60,000 subjects with migraine and 315,000 controls identified 38 susceptibility loci for migraine, with these loci enriched for genes expressed in vascular and smooth muscle tissues (9). Additional interpretation of these genetic data suggests a substantial over-representation of vascular-related pathways, specifically including morphogenic and developmental vascular pathways (10).

Despite this evidence, relatively few studies have examined the relationship between the anatomic structure of the cerebral vessels and migraine. Research on atherosclerotic plaque has demonstrated the role that vessel anatomy plays in the location of plaque formation, with shear stress and flow dynamics contributing to endothelial injury in specific anatomic regions (11). Given the predominance of aura symptoms localizing to the occipital cortex in migraine, as well as a reported predilection for subclinical infarctions in migraine to occur in the cerebellum, the vertebrobasilar system (VB) is of particular interest (12). Furthermore, multiple brain stem nuclei are involved in autonomic function, which is thought to be impaired in migraine, and studies have suggested that coupling of the hypothalamus to specific regions of the pons, thought to be a migraine generator site, may be involved during activation of migraine (10, 13). The VB system is anatomically unique in the body in that it represents two smaller vessels merging into a single larger vessel. Modeling of flow dynamics and shear stresses in the VB system has shown complex and highly variable patterns across common structural variations of VB anatomy (14). These structural variations appear to have direct clinical implications; for instance, basilar artery (BA) curvature has been associated with increased risk of brain stem infarction, even after adjusting for vascular risk factors, and infarctions appear to preferentially lateralize opposite curvature direction (15–17). To investigate the relationship between VB anatomy and migraine, we compared measures of vertebral artery (VA) asymmetry and BA curvature between migraine and control subjects.

## Materials and Methods

We performed the Anatomy and Cerebral Hemodynamic Evaluation of Migraine (ACHE-M) study, a prospective, observational, case-control study using high-resolution MR angiography, arterial spin-labeled (ASL) perfusion MRI, and structural MRI of the brain. The primary goal of the ACHE-M study was to compare the prevalence of an incomplete circle of Willis in migraine compared to control subjects. A detailed description of the study design and methodology has been published previously (8). Briefly, MWA, migraine without aura (MwoA), and control subjects between the ages of 25–50 years were enrolled in a 1:1:1 ratio.

Participants were recruited from the neurology clinic at the University of Pennsylvania and by advertisements in the wider community. Subjects were screened and examined by a single study neurologist and categorized using International Classification of Headache Disorders criteria (18). Enrollment took place between March 2008 and June 2012. Subjects with manifest vascular disease of any type were excluded, as were patient with prior neurovascular imaging studies to minimize the potential for referral bias.

Using 3 T MRI (Siemens Trio, Erlangen, Germany), the following sequences were obtained: T1-weighted localizer scans (TR/TE = 20/5 ms–8 mm slice–28 cm FOV–192 × 144 matrix–1 NEX), 3D volumetric T1-weighted MPRAGE scan (TR/TE = 1,620/3.87 ms–1 mm × 1 mm × 1 mm voxel size–25.6 cm FOV–256 × 256 matrix–1 NEX), and 3D time of flight MRA (TR/TE = 26/3.4 ms–1 mm slice/0.5 mm overlap–25 cm FOV–832 × 571 matrix). Resting, interictal cerebral blood flow (CBF) measurements were acquired using pseudocontinuous ASL perfusion MRI with gradient-echo echo-planar imaging readout (TR/TE = 4,000/18 ms–slice thickness/gap = 6 mm/1.2 mm–voxel resolution = 3.5 mm × 3.5 mm × 6 mm-labeling duration 1,125 ms–postlabeling delay time 1,200 ms). The labeling plane was positioned 90 mm below a 16 slice imaging slab. Thirty pairs of interleaved control and tag images were acquired.

**Figure 1:**
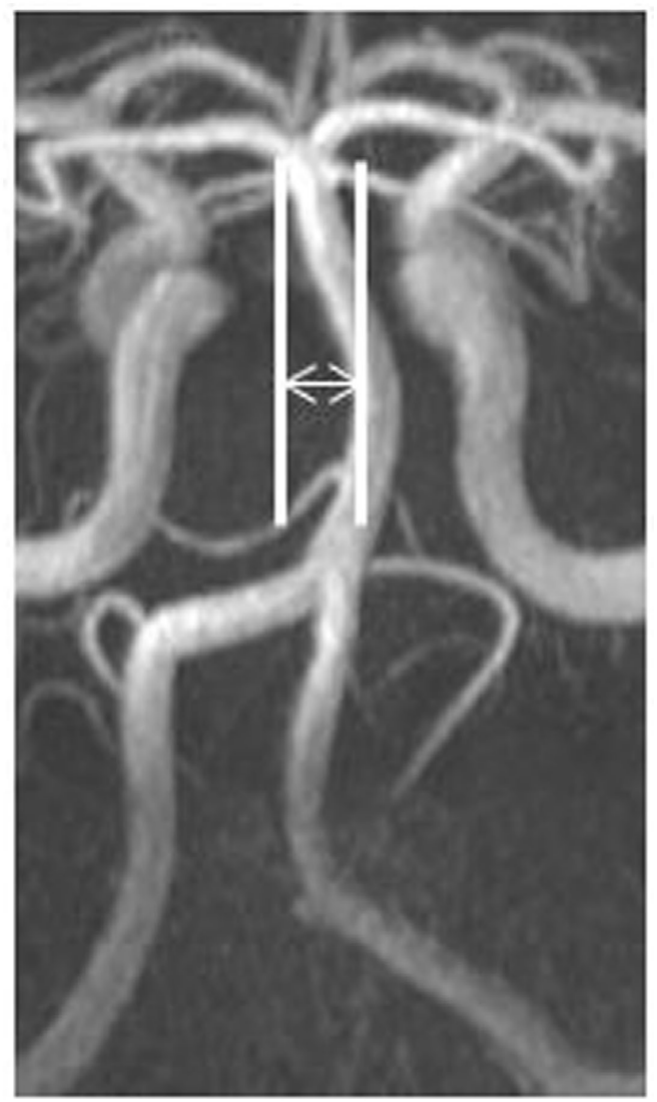
Measurement of basilar artery lateral displacement.

For the present analysis, a vascular neurologist blinded to migraine status and clinical data reviewed all MR angiograms and measured the following parameters: VA diameter measured just proximal to the vertebrobasilar junction, BA diameter measured at the midpoint of the BA, and BA total lateral displacement (defined as total distance between the furthest excursion of the BA to the left and right side of the brain stem). Vessel diameters were measured using MR angiography axial source images and displacement using reconstructed maximum intensity projection images. Figure 1 illustrates an example of BA displacement measurement. Measurement of regional CBF and of white matter lesion burden was performed as has previously been described (8, 19).

### Statistical Analysis

Baseline characteristics were analyzed using the chi-squared or Fisher’s exact test for dichotomous or categorical variables and the *t*-test or Wilcoxon ranked-sum tests for continuous variables as appropriate. Means and SDs or medians and interquartile ranges (IQRs) were calculated as appropriate. Associations between continuous variables were tested using multivariable linear regression, adjusting for factors that were associated in the univariate analysis at a significance level of *p* < 0.10. An association was considered significant if *p* < 0.05. As recommended by biostatistical experts, corrections for multiple comparisons were not performed (20). Statistical analyses were performed using JMP (Version 12, SAS Institute Inc., Cary, NC, USA).

## Results

A total of 178 subjects were enrolled in the ACHE-M study. Eight subjects were excluded from the original primary study prior to data analysis (four could not tolerate MRI due to claustrophobia, one had severe MRI artifact from permanent eye makeup, two were incorrectly enrolled despite exclusion criteria, and one had incorrect MRA field of view setting such that the circle of Willis was not visualized). An additional 8 subjects were excluded from the present analysis due to incomplete imaging of the vertebrobasilar system, leaving 162 subjects in the study cohort (52 control/52 MWA/58 MwoA). Baseline characteristics of subjects are described in Table 1. In the MWA cohort, 94% had typical binocular visual aura, 29% had monocular visual aura, 27% sensory aura, 10% motor weakness, 13% speech difficulty, and 40% described multiple types of aura symptoms. There were no subjects with basilar migraine.

**Table 1:** Characteristics of groups.

	Control (*n* = 52)	MWA (*n* = 52)	MwoA (*n* = 58)	*p*-Value MWA vs. control	*p*-Value MwoA vs. control
Age, mean ± SD (years)	32.2 ± 6.1	33.8 ± 7.1	33.7 ± 6.7	0.20	0.20
Female, *n* (%)	39 (75)	45 (87)	43 (74)	0.13	0.92
Systolic blood pressure, mean ± SD (mm Hg)	124 ± 16	126 ± 19	128 ± 19	0.51	0.26
Diastolic blood pressure, mean ± SD (mm Hg)	79 ± 9	82 ± 13	85 ± 13	0.17	0.02
Hypertension, *n* (%)	1 (2)	2 (4)	4 (7)	0.55	0.19
Diabetes, *n* (%)	0 (0)	1 (2)	0 (0)	0.24	–
Smoker, current or former, *n* (%)	11 (21)	12 (23)	23 (40)	0.81	0.03
Migraine duration, mean ± SD (years)		18.9 ± 9.8^a^	14.0 ± 8.7		
Migraine frequency, per month, median (IQR)		3 (1–5)^b^	2 (1–6)		
High white matter hyperintensity burden	17.3%	21.1%	24.1%	0.62	0.38
≥1 white matter hyperintensity	23%	27%	34%	0.65	0.18
PCA CBF (ml/100 g/min)^c^	54.8 ± 14.0	55.8 ± 14.5	55.8 ± 14.2	0.74	0.74
Basilar artery diameter, mean, SD (mm)	3.6 ± 0.6	3.6 ± 0.6	3.6 ± 0.6	0.70	0.94
Vertebral artery asymmetry, median, IQR (mm)	0.3 (0.2–0.8)	0.5 (0.1–1.0)	0.6 (0.1–1.1)	0.47	0.27
Basilar artery displacement, mean, SD (mm)	4.9 ± 3.1	6.3 ± 3.8	5.1 ± 3.0	0.055	0.76

*^a^p = 0.02 vs. MwoA*.

*^b^p = 0.61 vs. MwoA*.

*^c^Perfusion data were available for 149 of the subjects (47 control/49 MWA/52 MwoA)*.

*MWA, migraine with aura; MwoA, migraine without aura; IQR, interquartile range*.

**Figure 2:**
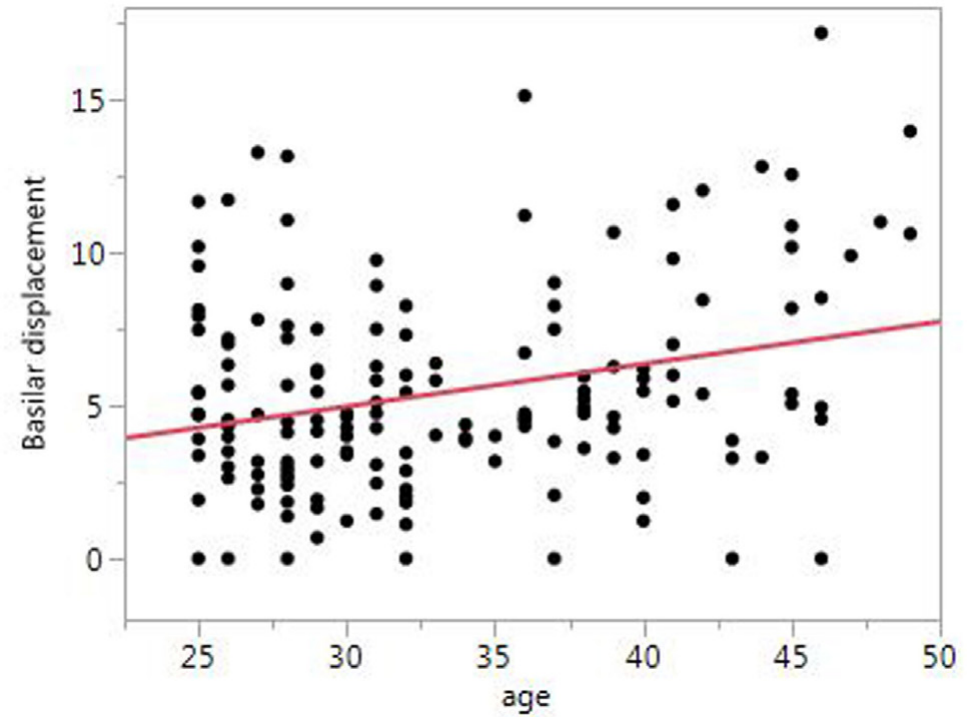
Relationship between basilar artery displacement and age.

**Figure 3:**
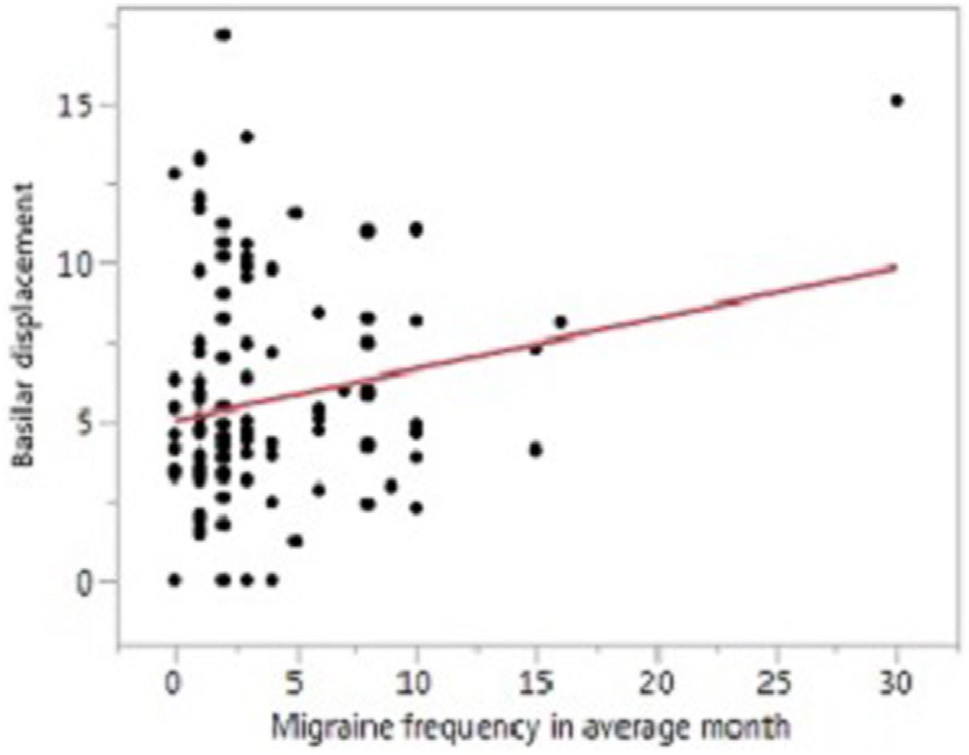
Relationship between basilar artery displacement and migraine frequency.

**Figure 4:**
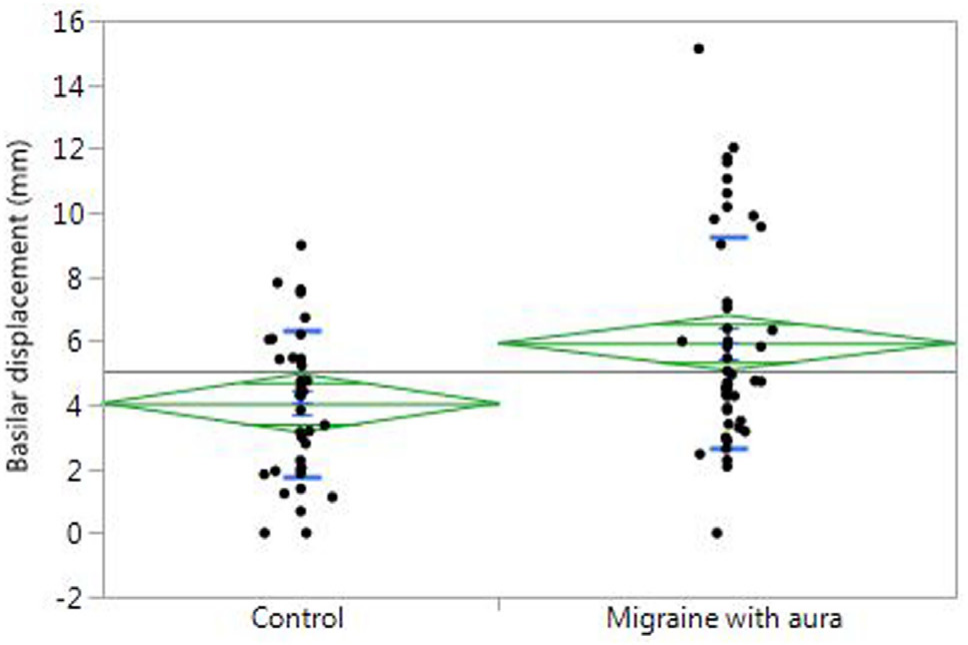
Basilar artery displacement in women with migraine with aura compared to controls.

In univariate analysis, VA asymmetry did not vary based on migraine status or other baseline characteristics. BA diameter was greater in men compared to women (3.9 vs. 3.5 mm, *p* < 0.001), but did not vary based on migraine status or other baseline factors. BA displacement was greater in men compared to women (6.6 vs. 5.1 mm, *p* = 0.02), and was greater with older age (*p* < 0.001, Figure 2) and with greater migraine frequency (*p* = 0.03, Figure 3). BA displacement was similar in MwoA (5.1 ± 3.0 mm) and controls (4.9 ± 3.1 mm), but tended to be greater in MWA subjects (6.3 ± 3.8 mm, *p* = 0.055 vs. controls). In women, MWA was associated with significantly greater BA displacement than controls (5.9 vs. 4.1 mm, *p* = 0.004), whereas there was no significant difference in men (8.4 vs. 7.6 mm, *p* = 0.73) (Figure 4). In analysis restricted to subjects with migraine, there was no significant relationship between BA displacement and number of years of migraine.

In multivariate analysis including age, sex, and headache status in the model, BA displacement was significantly greater in MWA subjects compared to controls (0.86 mm, 95% CI 0.14–1.57, *p* = 0.02), with older age (0.11 mm per year, 95% CI 0.04–0.19, *p* = 0.003), and in men compared to women (0.63 mm, 95% CI 0.01–1.25, *p* = 0.046). In multivariate analysis adjusted for age and sex but restricted to only subjects with migraine, BA displacement remained associated with increasing migraine frequency (0.17 mm per headache/month, 95%CI 0.03–0.31, *p* = 0.02). Additional analysis including measures of circle of Willis integrity (any circle of Willis variant, any posterior circulation variant or total circle of Willis variant burden) in the model did not substantively change the results.

For analysis of the association between posterior cerebral artery (PCA) territory CBF and basilar displacement, left and right PCA CBF did not differ so these were combined into a single measure of PCA CBF. There was no significant relationship between BA displacement and combined resting PCA CBF (*p* = 0.44). Resting bilateral PCA CBF was also not significantly different between the top quartile of BA displacement versus the bottom three quartiles (54.8 ± 15.1 vs. 55.7 ± 13.9 ml/100 g/min, *p* = 0.73) Subjects in the top quartile compared to bottom three quartiles of white matter lesion burden tended to have greater BA displacement (6.3 vs. 5.2 mm, *p* = 0.09), however, when adjusted for age, which is strongly associated with WMH load, this association was not significant (*p* = 0.43).

## Discussion

We found a significant correlation between BA curvature, quantified by measuring total lateral displacement of the BA, and MWA. Furthermore, patients with increased migraine frequency seemed to have greater degrees of BA displacement. The pathophysiologic mechanisms underlying these potential associations are uncertain. One possibility is that shear stress induced by the tortuous vessel results in endothelial activation increasing the probability of triggering cortical spreading depression in the downstream occipital lobes (1). An MRI study using computational fluid dynamics modeling demonstrated areas of higher peak wall shear stress in normal control subjects with BA variations associated with greater curvature (14). Another possibility is that BA curvature is a preexisting anatomic variant that causes local mechanical compressive injury to the brain stem and/or trigeminal nerve causing trigeminovascular activation increasing the likelihood of migraine. A similar phenomenon of local compression by aberrant vascular structures is known to underlie trigeminal neuralgia (21–23). However, if this were the case, one might expect an association with MwoA as well, which we did not find. A final possibility is that increased BA curvature is a result (rather than a cause) of recurrent migraine attacks. A high resolution MRA study has demonstrated intracranial arterial vasodilation in subjects during migraine with vasoconstriction following sumatriptan administration (24). Such a phenomenon might lead to repeated alterations in normal flow patterns in the BA causing uneven vascular remodeling over time which leads to increasing BA curvature. Our study unfortunately does not provide significant insight into which (if any) of these potential mechanisms might be operative. Longitudinal data on the time course of development of BA curvature would be extremely useful to help assess whether BA curvature is a causative contributor to migraine, or an effect of repeated migraine attacks.

In addition to the finding that BA displacement is associated with MWA, we also found that displacement increased with older age and was greater in men than women. This is concordant with prior data from the Secondary Prevention of Small Subcortical Stroke study demonstrating that vertebrobasilar ectasia, which strongly correlates with BA tortuosity, was greater with older age and in men (25). It is perhaps somewhat surprising that a correlation between age and BA displacement was seen in our study even with the relatively limited and younger age range of our subjects. This finding suggests that there may be a progressive component to BA curvature related to vascular remodeling over time, and that, given the very low prevalence of vascular risk factors in our cohort, this may be independent of traditional markers of atherosclerosis. We did not find any correlation between BA curvature and interictal PCA blood flow or white matter lesions, such that cerebral hypoperfusion seems unlikely to explain a link between BA curvature and migraine.

Our study has several limitations. First and foremost, this was a *post hoc* secondary analysis and thus should be considered hypothesis generating; confirmation in other cohorts is necessary. Second, measurement of the total lateral displacement of the BA, while reliable and easily determined, may not be the optimal measure of vascular tortuosity as it does not quantify other geometric factors which may impact flow dynamics, such as total length of the BA, angles of the inflow feeding VA, and outflow branching vessels. Third, the association between MWA and BA displacement was significant in women, but not men. This may be due to a lack of statistical power to detect a difference in men since the majority of our population was female (reflecting the population prevalence of migraine), or it may reflect biological differences in migraine mechanisms by sex.

In conclusion, our study supports a relationship between cerebrovascular structure, in this case BA curvature, and MWA. This association does not appear to be mediated by cerebral hypoperfusion. Further large, prospective studies are needed to confirm these findings. If replicated in future studies, further investigation of the link between migraine and BA anatomy might provide new insights into the physiological mechanisms underpinning migraine.

## Ethics Statement

The study was carried out in accordance with the recommendations of the University of Pennsylvania Institutional Review Board with written consent from all subjects. All subjects gave written informed consent in accordance with the Declaration of Helsinki. The protocol was approved by the University of Pennsylvania Institutional Review Board.

## Author Contributions

CZ and BC contributed to the study concept and design, analysis, and interpretation of data. JD and SK provided critical revision of the manuscript for important intellectual content. BC carried out acquisition of data and study supervision. All authors read and approved the final manuscript.

## Conflict of Interest Statement

The authors declare that the research was conducted in the absence of any commercial or financial relationships that could be construed as a potential conflict of interest. The reviewer FA and handling Editor declared their shared affiliation.
